# Molecular profiling and therapeutic tailoring to address disease heterogeneity in systemic lupus erythematosus

**DOI:** 10.1007/s10238-024-01484-z

**Published:** 2024-09-19

**Authors:** Abhibroto Karmakar, Uma Kumar, Smitha Prabhu, Vinod Ravindran, Shankar Prasad Nagaraju, Varashree Bolar Suryakanth, Mukhyaprana M. Prabhu, Subhradip Karmakar

**Affiliations:** 1 Department of General Medicine, Kasturba Medical College, Manipal, Manipal Academy Higher Education, Manipal, India; 2 Department of Rheumatology, All India Institute of Medical Sciences New Delhi, New Delhi, India; 3 Department of Dermatology, Kasturba Medical College, Manipal Academy Higher Education, Manipal, India; 4 Department of Rheumatology, Centre for Rheumatology, Kozhikode, Kerala India; 5 Department of Nephrology, Kasturba Medical College Manipal, Manipal Academy Higher Education, Manipal, India; 6 Department of Biochemistry, Kasturba Medical College Manipal, Manipal Academy Higher Education, Manipal, India; 7 Department of Biochemistry, All India Institute of Medical Sciences New Delhi, New Delhi, India

**Keywords:** Systemic lupus erythematosus, Multiomics, Personalized medicine, Single-cell transcriptomics, CAR-T-cell therapy

## Abstract

Systemic lupus erythematosus (SLE) is a chronic, heterogeneous, systemic autoimmune disease characterized by autoantibody production, complement activation, and immune complex deposition. SLE predominantly affects young, middle-aged, and child-bearing women with episodes of flare-up and remission, although it affects males at a much lower frequency (female: male; 7:1 to 15:1). Technological and molecular advancements have helped in patient stratification and improved patient prognosis, morbidity, and treatment regimens overall, impacting quality of life. Despite several attempts to comprehend the pathogenesis of SLE, knowledge about the precise molecular mechanisms underlying this disease is still lacking. The current treatment options for SLE are pragmatic and aim to develop composite biomarkers for daily practice, which necessitates the robust development of novel treatment strategies and drugs targeting specific responsive pathways. In this communication, we review and aim to explore emerging therapeutic modalities, including multiomics-based approaches, rational drug design, and CAR-T-cell-based immunotherapy, for the management of SLE.

## Introduction

Systemic lupus erythematosus (SLE) is a multifaceted disease and is well-known to have an unpredictable fluctuating course with periods of exacerbation and remission [1]. A significant number of patients with SLE have uncontrolled disease due to genetic and environmental factors, and this combined with adverse effects of treatment, contributes to multiorgan damage, leading to increased comorbidities and thereby impacting patients’ quality of life [2]. With the improved understanding of the pathophysiology and molecular mechanisms involved in the development of SLE, advances in personalized medicine are being made. The standard treatment regimens include various drugs, including glucocorticoids, antimalarials, immune suppressive, corticoids, and biologics. However, regimens vary among individuals and cause a substantial degree of damage. Recent therapeutic strategies have aimed to target novel evidence-based tailored strategies and molecular genotyping of patients [3, 4]. In this review, we discuss in detail the development and advancements in treatment strategies for lupus, including cellular-based therapy, such as chimeric antigen receptor (CAR-T) cells; targeting B cells, T cells, and small circulatory miRNAs; and targeting specific pathways, along with the use of precise transcriptomics, genomics, and peptide-based immunomodulators to aid and effectively manage SLE [5]. Additionally, all these therapies are under trial and not approved until now while studies are going on.

### *CAR*-T-cell therapy in SLE

**Fig. 1:**
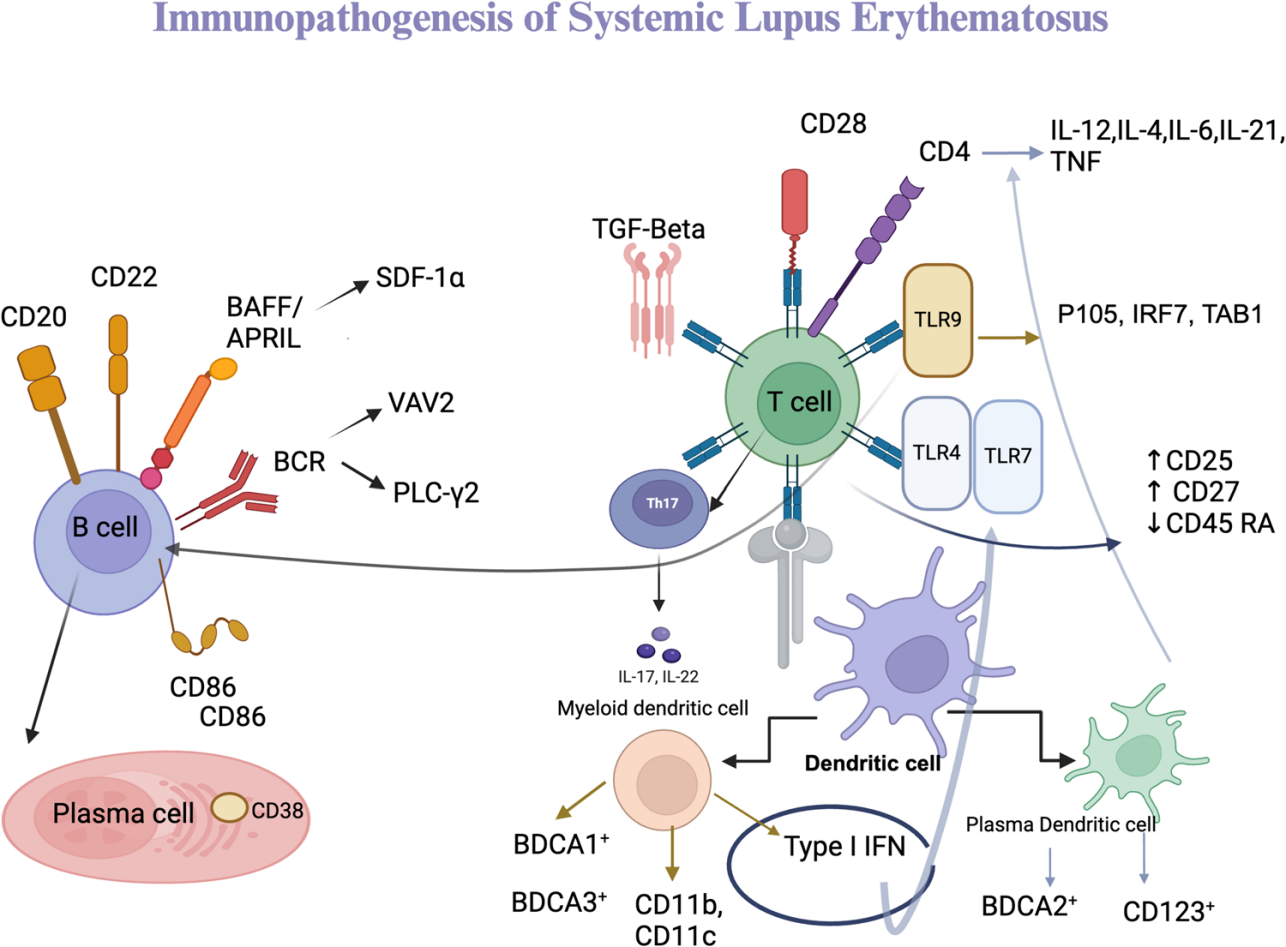
Immunopathogenesis of B cell, T cell, and dendritic cell in lupus. BAFF: B-cell activating factor, APRIL: proliferation-inducing ligand, BCR: B-cell receptor, VAV2: Vav guanine nucleotide exchange factor 2, PLC ý: Phospholipase C gamma 2, TGF-ß: Transforming growth factor beta, TLR: Toll-like receptor, IFN: Interferon, IRF: Interferon regulatory factor

Chimeric antigen receptor T (CAR-T) cells are engineered recombinant T cells used for treating CD19 + B-cell malignancies and are now widely used for treating various autoimmune diseases [6]. CAR-T cells integrating targeted CD19 + B cells have been introduced for the treatment of SLE patients, and understanding the immunopathogenesis of the disease is much needed (Fig. 1). Mackeson et al. studied five patients with treatment-refractory SLE and showed that patients with CD19-related SLE achieved drug-free remission after receiving CAR-T cells, which effectively deplete B cells. CAR-T-cell therapy is safe and sufficient functional CAR-T cells can be generated from patients with SLE. The breakdown of B-cell-mediated autoimmune SLE remains absent, even after B-cell reconstitution, and none of the patients developed a flare-up of SLE. One important benefit of using CAR-based therapy to treat autoimmune disorders is that it targets CD19, which is also expressed by plasma blast cells [7, 8]. For the first seven SLE patients receiving autologous CD19-directed CAR-T-cell therapy, long-term clinical effectiveness and safety data were obtained. All patients experienced long-lasting drug-free remission and were disease-free for more than 22 months, based on evidence. Patients with severe SLE may benefit from CD19 CAR-T-cell therapy, which can prevent the disease [9]. Advancements in CAR-T-cell technology potentially provide better and more promising new avenues as effective treatment options for many autoimmune diseases [10].

The effectiveness of Kansal et al. investigated a CD19-targeted CAR-T-cell strategy and demonstrated the possibility of using this approach in disorders such as lupus. CAR-T cells targeting CD19 are more effective than antibody-mediated cytotoxic agents. B cells are efficiently depleted when CD19-targeted CAR-T cells deplete them without needing any other accessory cell type. Additionally, activated CD19 is expressed on B cells, such as developing plasma blasts and early plasma cells that target B cells stimulated by autoantigens and oversee the production of autoantibodies. Simultaneously, by verifying that the CAR-T cells remained functional for several months and depleting the transferred autologous CD19 + B cells, cell-based therapies ensure that CD19 + B cells are depleted from various tissues to which CAR-T cells can access, such as the spleen and bone marrow, as shown in a mouse model [11]. Additionally, the Food and Drug Administration has authorized CAR-T-cell therapy and granted the Fast Track designation to CABA-201 for treating systemic lupus erythematosus (SLE) and lupus nephritis. CABA-201 is a completely human CD19 CAR-T-cell line in which 4-1BB depletes CD19-positive B lymphocytes, causing disease inactivity in patients with lupus nephritis and SLE [12, 13].

Some studies show promising results in CAR-T-cell therapy, including Li et.al patient with refractory thrombocytopenia of SLE when treated with CAR-T cells, show complete elimination of circulating CD19 B cells within a month [14]. In pediatric lupus nephritis Anti-CD19 CAR-T cells, therapy achieved sustained remission a gradual decrease in inflammatory cytokines IL-6 and TNFα, along with the significant drop in SLE-associated antibodies in five out of six patients [15]. While experimental therapies offer hope for patients, it is crucial to approach them with realistic expectations. Rigorous and well-designed clinical trials are essential to determine their true value in assessing safety and efficacy and identifying optimal treatment approaches. Very few clinical trials 25 studies out of which 64% of studies conducted by China have been registered under clinical trials for CAR-T-cell therapy in SLE. An open, Phase I clinical trial conducted in China has been conducted in moderate to severe SLE [16]. Similarly GC012F injection (CD19-BCMA CAR-T cells) in patients with refractory systemic lupus erythematosus for dual target CAR-T-cell treatment has been explored [17]. BCMA-CD19 cCAR-T cells therapy in patients with relapsed and/or refractory SLE [18]. Another trial including cutaneous lupus is done using BRL-301 in refractory SLE cases [19]. Trials in childhood lupus to understand the therapeutic efficacy of anti-CD19 CAR-T cells are being conducted [20].

Additionally, a CARLYSE a phase I study of obecabtagene autoleucel (obe-cel), autologous T cells engineered with a chimeric antigen receptor (CAR) targeting CD19, with severe, refractory systemic lupus erythematosus (SLE) is under registered trial [21]. The experimental technique has potential risk, limitation and challenges: Mild cytokine syndrome has been seen in several observations including Mackensen and group series of five patients. Wang et.al using BCMA-CD19 compound chimeric antigen receptor T cells (cCAR) dual technique as well as case series conducted by Muller et, al shown grade 1 cytokine release syndrome proves CD19 CAR-T-cell transfer is feasible, tolerable, and highly effective in SLE; however, long-term consequences need to study more [22–24]. Immune effector cell-associated neurotoxicity syndrome (ICANS) is a potentially life-threatening neurotoxicity that commonly occurs with CAR-T-cell therapy [25]. However, no ICANS was observed and CRS observed was mild (grade I) in SLE patient [22].

The status of the technique raises questions regarding the feasibility of engineering specific T cells for every patient including significant toxicities like cytokine release syndrome (CRS), neurotoxicity, immunosuppression, and cytopenia challenges [26, 27]. The complex manufacturing process results in high treatment costs as well as limited accessibility to therapy for many patients, and responses differ due to polygenicity of the disease. Long-term outcomes and safety profiles are still under investigations [28]. The main challenges that arise with the selection of the patients with treatment resistance, severity of the organ damage, and disease activity for the procedural of the therapy should be carefully monitored along with safety, efficacy, and toxicity [29]. The technical complexity, accessibility, and cost persist overall challenges of CAR-T-cell therapy in SLE. FDA-approved CAR-T therapies currently cost US$350,000–500,000, with problems down the road around EUR 350.000 [30, 31]. The complexity lies in the manufacturing process, development, quality control, and the requirements for industrial scaling [32]. Contemplate is that obtaining informed consent from patients is crucial, especially given the experimental nature of CAR-T-cell therapy. The long-term consequences of CAR-T-cell therapy in SLE, including the potential for unexpected side effects or long-term changes in immune function, require careful monitoring and ethical consideration. The production and administration of CAR-T cells can be expensive due to the complex manufacturing process and specialized care requirement. Traditional treatments for SLE often involve a combination of medications, including immunosuppressants and corticosteroids. These treatments can have significant side effects and may not always achieve long-term remission [33]. CAR-T-cell therapy could offer several potential advantages over traditional treatments; CAR-T-cell therapy induces longer-lasting remission than traditional treatments, and patients may experience fewer adverse events. The ability to target specific antigens like CD19 on B cells provides a high degree of specificity, reducing the risk of off-target effects [34]. Large-scale production of CAR-T cells for a broader patient population remains a challenge. However, ongoing research and technological advancements are addressing this issue [35]. CAR-T-cell therapy is currently expensive due to personalized nature of the treatment. However, as manufacturing costs decrease and insurance coverage expands, the treatment may become more accessible. As the technology matures and costs decrease, CAR-T-cell therapy may become a viable treatment option for patients with SLE. Dias et al. discussed the manufacturing process, development, quality control, and the requirements for industrial scaling [32]. Obtaining informed consent from patients is crucial, especially given the experimental nature of CAR-T-cell therapy and the potential benefits, risks, and uncertainties associated with the treatment. The long-term consequences of CAR-T-cell therapy in SLE, including the potential for unexpected side effects or long-term changes in immune function, require careful monitoring and ethical consideration [36]. CAR-T-cell therapy could offer several potential advantages over traditional treatments: CAR-T cells directly target B cells, induce longer-lasting remission, and reduce side effects, potentially leading to more effective treatment. The high upfront costs and specialized infrastructure required for CAR-T-cell therapy can make it challenging to implement in resource-limited settings. However, several strategies could be explored to improve accessibility: Governments, healthcare providers, and pharmaceutical companies could collaborate to develop cost-sharing programs that make CAR-T-cell therapy more affordable. Establishing regional manufacturing centers could reduce the cost of producing CAR-T cells. Supporting research into CAR-T-cell therapy in resource-limited settings can help identify more cost-effective and accessible treatment options [5]. CAR-T-cell therapy has shown significant promise in other hematological malignancies. The technology is advancing rapidly, making it more feasible for application in SLE. Large-scale production of CAR-T cells for a broader patient population remains a challenge. However, ongoing research and technological advancements are addressing this issue. Presently, there is a lack of extensive long-term data regarding the efficacy of CAR-T-cell therapy. Clinical applications are mainly in the investigative phase with few clinical trials, requiring ongoing assessment of safety and long-term results. The practical limitations of producing patient-specific cell products hinder the technology application [37, 38].

### Single-cell transcriptomics in SLE

Single-cell RNA sequencing (scRNA-seq) technology helps to understand and reveal individual cells heterogeneity and complexity. Since their discovery in 2009, they have helped in the subgrouping of different cell types and better understanding of the composition and interaction of cells [39]. The use of computational and experimental scRNA-seq technology for treating many diseases, especially in personalized medicine, has increased due to the development of high-resolution cell catalogs for identifying better therapeutic targets for any disease [40]. Single-cell technologies have enabled molecular stratification through high-throughput cell profiling and open chromatin accessibility, spatial information, and protein surface expression [41]. In recent years, multiplex single-cell RNA sequencing (mux-seq) has been used to profile peripheral blood mononuclear cells to elucidate genes and transcriptional signatures linked to systemic lupus erythematosus (SLE) and to specifically define their cellular makeup [42]. The recent review by Perez et al. revealed specific cell types and their genetic associations with lupus. Using multiplexed single-cell RNA sequencing, they analyzed over 1.2 million peripheral blood mononuclear cells (162 patients, 99 controls) and demonstrated a decrease in naïve CD4^+^ T cells with an increase in type 1 interferon-stimulated genes (ISGs) in monocytes. They further stratified patients into two molecular subgroups, linking SLE-associated mutations to cell type-specific cis-expression quantitative trait loci, and integrated rich genotyping data identifying transcriptional signatures and genetic variants associated with SLE [43]. Dunlap and colleagues presented a comprehensive single-cell RNA-Seq profile of T and NK cell types found in cutaneous lupus patient lesioned and nonlesional skin biopsies, demonstrating increased expression of IFN-simulated genes. These findings allow for a cross-tissue comparison revealing striking variations in the makeup and activity of T/NK cells in various lupus tissues, constituting the first comprehensive transcriptome investigation of T and NK cells in cutaneous lupus at the single-cell level [44]. Evan et al., in their recent review, showed that type I interferon is involved in the pathogenesis of lupus. More specifically, tubular cells upregulate the type I interferon signature in addition to infiltrating cells. Additionally, single-cell transcriptomics revealed that pathways targeting ECM proteins involved in the fibrotic response are highly upregulated in nonresponse patients [45, 46].

Single-cell transcriptomics, along with ATAC-Seq (for identifying assessable chromatin), has revolutionized molecular biology [47]. Guo et al. revealed the role of Treg cells in transcriptional dysfunction through ATAC-seq and single-cell transcriptome sequencing (scRNA-seq) of peripheral CD4 + T cells from 72 patients with SLE and 30 healthy controls. Specifically, two Treg subpopulations, the CCR7 and CD74 Treg subgroups, are associated with type I interferon-induced dysfunction in SLE patients, suggesting that specific pathways in lupus need to be targeted. Tregs represent a new target for drugs for the treatment of lupus [48]. Another area is single-cell proteomics (SCP), which is at the edge of revolutionizing the field of single-cell biology, and various studies have shown that the regulation of translation is more stable than that at the transcriptome level, especially where the heterogeneity of diseases is like that of lupus [49]. Bulk proteomics lacks sensitivity in detecting rare cells, which can only be detected by the SCP technique. This technique has enabled us to understand posttranslational modifications and integrate transcriptomic data. There have been improvements in SCP techniques, but high-throughput, automated, reliable, and scalable technologies for SCP investigation are still being developed [50, 51]. However, single-cell omics technologies will eventually offer a genuinely holistic and all-encompassing perspective of single-cell biology and aid in unraveling the enigma of lupus at the single-cell level [52]. The current scenario of single-cell RNA sequencing (scRNA-seq) helps in understanding transcriptionally defined subpopulations within major cell types, including monocytes, CD4 + and CD8 + T cells, natural killer cells, and plasmacytoid dendrites, at different degrees of disease activity [53]. Despite its potential, the application of scRNA-seq to population cohorts has been limited by low sample throughput, high cost, and susceptibility to technical variability. The future of single-cell transcriptomics makes understanding disease heterogeneity easier, but understanding the computational algorithm, cost and scalability still greatly hinder its use in daily practice. Single-cell transcriptomics (scRNA-seq) is a powerful tool for understanding the heterogeneity of diseases like systemic lupus erythematosus (SLE). However, the direct application of scRNA-seq in clinical settings for SLE patients is still immature and undersized. The main challenges or potential risks lie in capturing the functional state of immune cells at different disease activities along with data acquisition. Longitudinal studies need to be done addressing the clinical heterogeneity of the disease. A translational challenge in identifying clinically actionable biomarkers and therapeutic targets is complex and requires further validation. Noise in single-cell RNA seq data is high compared to bulk transcriptomics. Bioinformatics analysis of scRNA-seq data is still challenging [54] and computational pipelines for handling raw data files remain limited [55]. SLE samples, especially from specific tissues at different disease activities, can be difficult to procure**.** The choice of method droplet-based versus plate-based impact data quality and analysis [56]. Epigenetic modifications play a significant role in SLE but are often not captured by scRNA-seq alone. Bridging the gap between scRNA-seq findings and clinically relevant biomarkers or therapeutic targets can be challenging [57].

SCT examining gene expression on a single-cell level offers unprecedented understanding of cellular variation, immune cell types, and causes of disease. Long-term outcomes may involve categorizing disease subtypes or identifying patient-specific biomarkers, resulting in more precise and efficient therapies. Long-term advancement will aid in forecasting lupus flares and tracking response to treatment, discovering new targets and pathways to speed up the creation of novel treatments [58, 59]. SCT relatively expensive technology due to the high cost of sequencing and computational analysis. However, technological advancements and economies of scale have led to a gradual decrease in costs. Further cost reduction is expected as the technology becomes more widely adopted and standardized [60]. FDA approval may be necessary for the use of single-cell transcriptomics as a diagnostic or prognostic tool hinderance to the regulatory factors in single-cell transcriptomics. The management of patient-level genomic data needs to follow strict privacy regulations. The analysis of extensive single-cell transcriptomic datasets necessitates specific bioinformatics tools and skills. This could pose a bottleneck in environments where resources are limited. Ensuring consistent quality checks, standardization consistency and comparability in research through the use of standardized procedures for sample collection, processing, and data analysis [61]. Patients must be provided with adequate information about the single-cell transcriptomics, including utilizing their personal genetic information as well. Equitable research in single-cell transcriptomics should strive to include a variety of patient populations. Starting a single-cell transcriptomics laboratory can require a substantial initial investment, particularly in areas with limited resources. In the long run, potential benefits like better diagnosis, prognosis, and treatment results may compensate for the initial expenses. In comparison with conventional treatments: The cost-effectiveness of single-cell transcriptomics in contrast to traditional SLE treatments (such as immunosuppressants and biologics) will vary based on factors like the specific use, result precision, and personalized treatment potential. Lack of essential infrastructure (such as high-throughput sequencing bioinformatics tools) is a significant problem in areas with limited resources [62]. According to Rao et al., single-cell genomics data can play a role in shaping clinical decisions. Their research illustrated the use of single-cell RNA sequencing in studying renal inflammation in lupus nephritis [63]. Likewise, Sande et al. demonstrated that observing diseased tissues throughout treatment in a longitudinal study can offer physicians a more direct and mechanistic understanding of how patients respond to treatment [64].

### Epitranscriptomics in SLE: Role of RNA modification in SLE pathogenesis

**Fig. 2:**
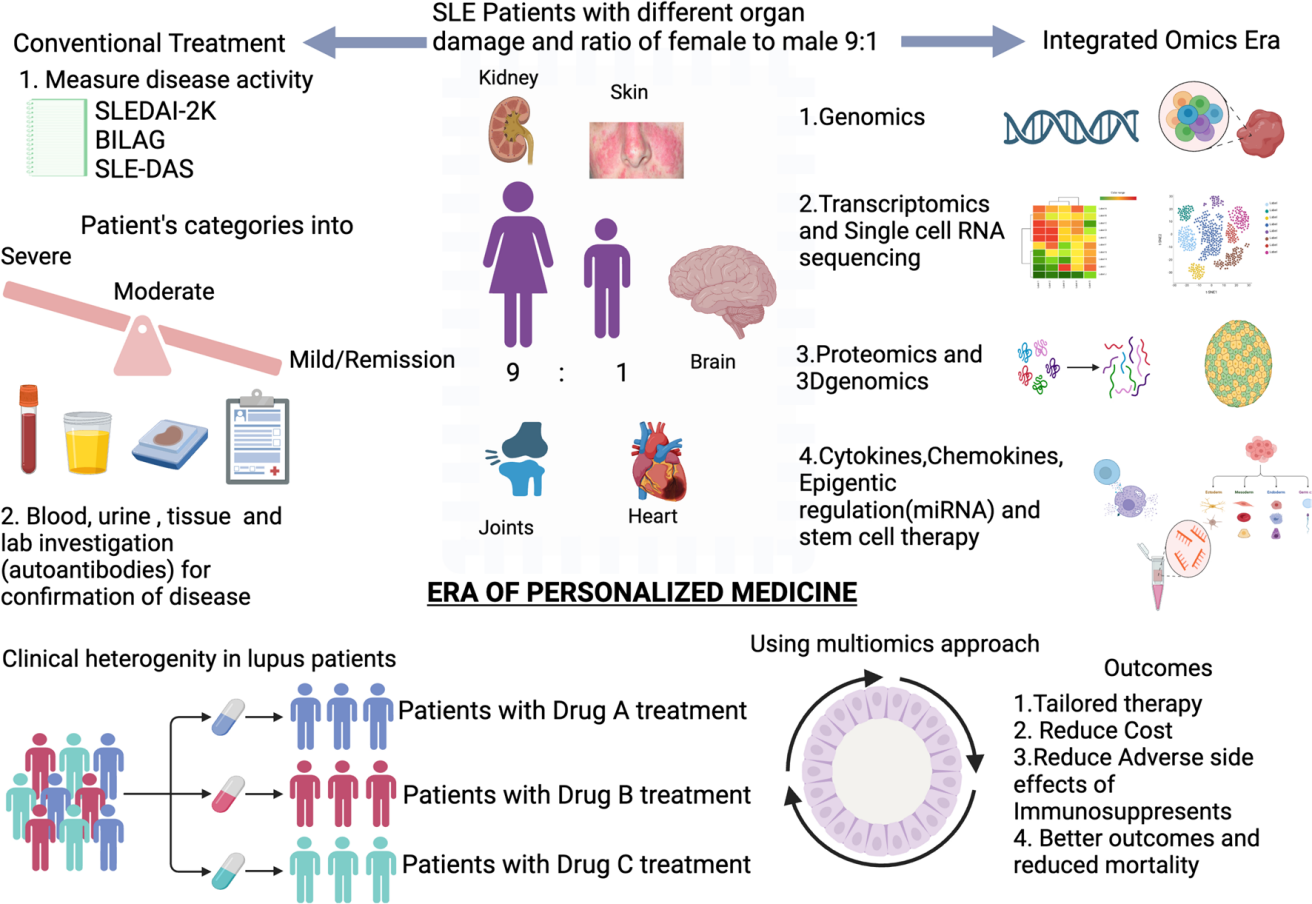
Illustrate SLE patients with different clinical manifestations. The conventional approach to treating the disease versus the era of molecular and personalized medicine

The etiology of SLE is not well-known and includes genetic, epigenetic, and environmental factors that dysregulate the immune system an epigenetic mechanism is one of the main players in autoimmune disorders. Significant RNA base line m6A modification has been observed in patients with SLE [65, 66]. Several epigenetic compounds with therapeutic potential are under clinical trial for malignancy, but their beneficial role in autoimmunity is less explored due to adverse side effects. In recent years, we have elucidated the role of epigenetics and its correlation in understanding the pathogenesis of this disease [67]. Epigenetic modifications include DNA methylation, chromatin remodeling through histone modifications, and noncoding RNAs—affecting gene expression without affecting the genomic sequence [68]. N4-acetylcysteine has been reported to be an important epigenetic modifier in systemic lupus erythematosus (SLE) [57]. The ac4C distribution in mRNA transcripts was substantially conserved and enriched in mRNA coding sequence areas by transcriptome-wide ac4C profiling through ac4C-RIP-Seq in CD4^+^ T cells from SLE patients. The targeted signaling pathways include NF-beta and ROS-induced cellular signaling pathways. Additionally, the unique ac^4^C-related transcripts revealed new dysregulated ac4C mRNAs that may be translated in lupus CD4 + T cells and linked to important immunological and inflammatory responses. Given the importance of ac4C mRNA alterations and transcriptional relevance, ac4C is a possible treatment target in the pathophysiology of SLE. Single-cell transcriptome profiling revealed an exhausted regulatory CD4 + T-cell subset in systemic lupus erythematosus [69, 70]. Luo et al. and colleagues studied N6-methyladenosine epigenetic modifications in patients with SLE. Their study indicated that the pathophysiology of SLE is influenced by the mRNA level of ALKBH5 (writer) in peripheral blood [71]. SLE may result from altered three-dimensional (3D) genomic architecture and long-range interactions that alter gene expression by rewiring intrachromosomal connections in a tissue- or cell-specific way. Along with the disease activity of SLE, patients' T cells display unique 3D chromosomal arrangements [72, 73]. Histone alterations include determining chromosomal loops linked to the action of SLE and genes with variable expression. Furthermore, the overexpression of the interferon pathway gene is attributed to the transcription factor SPI1, whose motif is in the altered loop in SLE [74, 75]. 3D genome structural changes linked to the development of SLE provide new information for examining the connections between chromosomal structure and the regulation of gene expression in SLE [76]. Epitranscriptomics has revolutionized the field of cancer since its discovery in 2011[77]. This technology has increased the ability to map and engineer RNA modifications and understand RNA biology and methylation states in patients with lupus. The future of the technique lies in therapeutic and target development in lupus using mapping, RNA modification, and a supervised machine-learning approach. The conventional approach to treating SLE is now changing and the use of a modern multiomics approach to come as personalized therapy in lupus (Fig. 2). Epitranscriptomic modifications are diverse and complex, making it challenging to decipher their exact roles in disease pathogenesis. Detecting and quantifying subtle epitranscriptomic modifications requires highly sensitive and specific techniques while advancements have been made, challenges remain in accurately measuring these modifications, especially at low abundance quality and integrity are critical for epitranscriptomics studies(81) [78]. Developing drugs that specifically targeted therapy for one type of RNA modification without affecting others is difficult [79]. Epitranscriptomic data can reveal sensitive genetic information, necessitating robust data protection measures [80]. Epitranscriptomic modifications are dynamic and can change rapidly in response to cellular signals and environmental cues. This makes capturing the full spectrum of modifications in a single snapshot challenging. Identifying epitranscriptomic biomarkers that are universally relevant to all SLE patients is challenging. While epitranscriptomic modifications offer potential therapeutic targets, translating basic research findings into effective clinical interventions can be a significant hurdle [81]. In a review, Adams and Shao noted that pharmaceuticals can reverse epigenetic modifications, making targeted therapy a viable option for treating SLE**.** Nevertheless, applying SLE epigenetics to precision medicine and finding therapeutic targets is difficult because of the widespread nature of epigenetic changes [57]. Thus, it is difficult to anticipate therapeutic results, which could be linked to adverse side effects. However, epigenetic abnormalities have been suggested as tools for diagnosing or predicting diseases [82]. Epitranscriptomics offers valuable insights into the pathogenesis of SLE, despite being a new field that requires long-term results to be observed. Wardowska reported epigenetic changes in antigen-presenting cells in SLE [83]. The technology and expertise needed for epitranscriptomics research can be costly. Mubarak et al. summarized key advances in transcriptomics and epitranscriptomics for precision and personalized medicine, with techniques mainly focused on research institutions and specialized laboratories [84]. Efforts are being made to make epitranscriptomics more available to clinicians and patients, though obstacles persist.

### Role of miRNA in SLE

Despite many attempts to comprehend the pathogenesis of SLE, there is still a lack of adequate knowledge about the precise mechanisms underlying the disease and to develop effective therapies for SLE patients. MicroRNAs (miRNAs) have been implicated in the pathogenesis of SLE in recent studies. miRNA dysfunction leads to autoimmunity. miRNA-mediated changes in B cells and T cells lead to the pathogenesis of SLE [85]. The expression of different miRNAs, such as miR-126, miR-21, miR-146a, miR-155, and miR-1246, is altered by epigenetic modifications, cell subset differentiation, B-cell hyperactivity, and autoantibody production [86].

miR-146a gene polymorphisms across populations have been identified as the genetic basis of SLE [87]. Distinct miRNAs are differentially expressed in both SLE mouse models and patients. miRNAs are important targeting molecules in SLE. MicroRNAs are small, noncoding RNAs that regulate gene expression at the transcriptional and translational levels. They play a crucial role in developing the immune system and regulate both the innate and adaptive immune systems. Altered expression of miRNAs is observed in autoimmune diseases such as SLE. miRNAs can bind to multiple mRNA targets, leading to unintended consequences. Manipulating miRNA expression could potentially exacerbate autoimmune responses in SLE [88]. Considering this, miRNAs have become an area of interest owing to their contributory role in disease pathogenesis. In SLE, both the innate and adaptive immune systems are activated, and specific miRNAs are linked to key processes involved in both processes. Some of these processes include interference in type 1 interferon (IFN)-signaling pathway (miR-146a, miR-155), DNA hypomethylation in T cells (miR-21, miR-126, miR-148a), inflammatory chemokine pathway (miR-125a), neutrophil development and function (miR-125a, miR-223, miR-451a), B-cell hyperstimulation and T-cell overactivation (miR-142-3p/5p) (16), the induction of regulatory T cells (miR-16), and the regulation of myeloid cell development (miR-223) [49, 50]. Significantly elevated levels of miR-199a-3p were discovered in lupus patients, and these levels were found to correlate with clinical features such as low C3 levels, positive anti-dsDNA antibodies, high ESR levels, active lupus nephritis, and active disease activity [89]. Zheng et al. demonstrated for the first time the differentially expressed circulatory RNAs in PBMCs and plasma. They established that the circulatory miRNA network may be a useful diagnostic biomarker for lupus [90]. Effective delivery of miRNA-based therapeutics to target tissues remains a significant hurdle. Inefficient delivery can reduce therapeutic efficacy and increase the risk of side effects [91]. The long-term safety profile of miRNA-based therapies is still under investigation. Potential long-term side effects cannot be ruled out. miRNA expression levels can change dynamically during SLE, making identifying consistent biomarkers or therapeutic targets challenging [92]. External factors such as stress, infections, and medications can also influence miRNA expression, adding to the system's complexity. Technical variability in sample collection, processing, and storage can introduce noise into miRNA expression data, affecting the reliability of findings. Accurate normalization of miRNA expression data is crucial for reliable comparisons, but it can be challenging due to the lack of universally accepted reference genes. Despite promising preclinical findings, there is a lack of large-scale clinical trials evaluating the therapeutic potential of miRNAs in SLE [93]. The development of miRNA-based therapies faces regulatory hurdles due to their novel nature and potential risks. Exosomal miRNAs, such as miR-21 and miR-15, could be useful biomarkers for detecting SLE and LN [94]. Despite being in its early stages, miRNA-based treatments have seen limited progress in clinical development, with none advancing to phase III trials and some being terminated because of toxicity concerns. Major obstacles involve achieving miRNA sensitivity, specificity, and selectivity toward their desired targets, reducing immunogenic responses and off-target effects, creating improved techniques for targeted transportation, and identifying the best dosage for therapeutic effectiveness with minimal side effects. Moreover, the clinical application of miRNAs is constrained by the incomplete knowledge about their specific functions [95]. Despite these limitations, ongoing research continues to explore the potential of miRNAs as diagnostic biomarkers and therapeutic targets in SLE. Overcoming these challenges will be essential for translating miRNA-based research into clinical practice. miRNA has not been used as a therapeutic in patients, so it will be too early to comment on long-term results. Kapoor et al. in gastric cancer found miRNA to be cost-effective in screening intervention [96]. Targeting dysregulated miRNAs could be a potential therapeutic approach for SLE involving using miRNA mimics miRNA inhibitors [97]. However, more research is needed to understand the specific roles of individual miRNAs in SLE and to develop safe and effective miRNA-based therapies. In the future, miRNAs will show promising results in various clinical applications for treating lupus, but there are potential limitations and adverse effects associated with their use. More detailed studies on the clinical utility of miRNAs in the diagnosis, detection of flare-ups and remission, and specific targeted treatment of lupus are needed.

### Stem cell therapy in SLE: future possibility

The current treatment for SLE involves treating patients with immunosuppressive agents, which have improved mortality but have failed to prevent flares. Therefore, alternative treatment options, such as stem cell therapy, can be approachable [98]. Stem cell treatment has been demonstrated to be a viable therapeutic method for treating individuals with refractory SLE in several animal studies and human trials. Stem cells modulate the innate and adaptive immune systems by regulating the origin and fate of the cells. These findings suggest that stem cells are promising potential therapeutic targets for treating lupus. The potential risks associated with stem cell therapy includes a majority of cases of relapse; Jayne et.al in 2004 showed significant relapse and the mortality of the patients was high [99, 100]. A study conducted by Jun Liang et al. reported significantly reduced disease activity in 15 Lupus patients treated with allogeneic stem cell therapy at the time of active disease. The percentage of peripheral blood regulatory T cells, renal function, and serological characteristics (antinuclear antibodies and anti-double-stranded DNA, or anti-dsDNA) were used to assess patient outcomes using the SLEDAI [101]. Yuan et al. demonstrated the process by which dendritic cells employ mesenchymal stem cells and examined serum FLT3L levels, which decreased substantially in SLE patients, and peripheral tolerogenic CD1c + dendritic cells (DCs). Consequently, it has been proposed that transplanting allogeneic umbilical cord-derived MSCs (UC-MSCs) considerably stimulates interferon-γ by upregulating serum FLT3L and peripheral blood in CD1c + DCs via activation of the JAK/STAT signaling pathway [102]. Cheng et al. showed an increase in CD4 + T-cell senescence through miR-199a-5p using mesenchymal stem cells as one of the mechanisms to alleviate lupus in a mouse model [103]. Autologous hematopoietic stem cell transplantation for long-term remission in SLE patients can be achieved by modifying the immune system, taking into consideration transplant-related mortality and the development of other comorbidities and secondary autoimmune diseases [104]. The short-term complications include infections including both bacterial, fungal and viral mucormycosis, cytomegalovirus, herpes zoster as well as methicillin-resistant *Staphylococcus aureus* (MRSA) endocarditis study done by Burt et al. [105]. A recent case study highlighted about chronic graft-versus-host disease Goeser et al. showed in pediatric lupus after hematopoietic stem cell transplantation [106]. Another study showed the incidence of grades II-IV acute GvHD was reported in which 53 out of 128 patients developed acute GvHD along with the case of new malignancy in one patient [107]. While promising, stem cell therapy for SLE is still in its early stages, and its long-term effectiveness is not fully established presents with a wide range of symptoms and severity, making it difficult to develop a one-size-fits-all stem cell therapy. Li et al. talked about mesenchymal stem cells being used in early clinical stages. Furthermore, MSC treatment enhances SLE but does not offer a complete cure. In a different study, it is still unclear whether MSCs are effective in treating LN, and potential advancements in stem cell science and specific mechanisms of action are needed for LN treatment confirmation in the future [108]. The effectiveness of stem cell therapy may vary significantly between patients due to genetic differences and disease progression. Introducing new cells into the body could potentially trigger an immune response and exacerbate SLE symptoms. Ensuring that stem cells reach the affected tissues and survive in the body can be challenging [109]. The ability of stem cells to integrate into the host environment and exert their therapeutic effects is crucial. Stem cell therapy can sometimes lead to immune-related side effects, such as graft-versus-host disease. The long-term safety and efficacy of stem cell therapy for SLE are still being investigated. The use of embryonic or fetal stem cells raises ethical concerns [110]. The average treatment cost for stem cell therapy is $4,000 USD to $8,000 USD**.** The cost is relatively high due to the complex cell conditioning and the need for high doses of immunosuppressive drugs [111]. MSCT in SLE patients were monitored longitudinally revealing that MSCT can enhance hematologic issues like leukocytopenia, thrombocytopenia, and anemia [112]. Only a few specialized centers in the world offer stem cell transplants for SLE with minimal insurance coverage additionally access to the specialized centers may be limited for patients in certain regions. Stem cell therapy for SLE would likely require rigorous clinical trials to establish safety and efficacy obtaining approval from FDA. Monitoring patients for long-term outcomes and potential side effects is essential. Logistical consideration, including cell source, processing, and accessibility particularly in resource-limited settings, is a significant challenge. Ethical guidelines for stem cell research and patients informed consent, understanding the potential risks and benefits of the therapy for long-term outcomes [113]. Stem cell therapy can be expensive due to the cost of cell isolation, processing, and transplantation. However, if proven to be effective in managing, it could potentially reduce long-term healthcare costs associated with the disease. Stem cell therapy may offer potential advantages in terms of efficacy and side effects. In resource-limited settings, affordable alternatives, collaborations, and patient priority will maximize the benefit. HSCT relapse and infection after transplantation are the major drawbacks of stem cell therapy. Stem cells can be used as an alternative therapy that has drastic effects on the immune profile of SLE animal models and patients. Future research should focus on understanding the different stem cell types involved and their pleiotropic effects on lupus [114, 115].

### Therapeutic peptides in SLE: new modalities

Numerous peptides have shown great promise in the treatment of systemic lupus erythematosus (SLE) in various studies. The rationale for their use is justified by their immunomodulating mechanism rather than their immunosuppressive nature. These materials are good candidates because of their low toxicity, minimal side effects, target selectivity, and cost-effectiveness. Currently, three therapeutic peptides have been tested in clinical settings and are undergoing clinical trials. They can be utilized in managing SLE flares and special subgroups of patients by combining them with small peptides and vaccines with highly efficient pharmacological effects [116, 117]. Although various peptides have entered phase II and III clinical studies and had primary results, P140, the CDR1-based peptide, and AMG623 have failed to meet the primary objectives but have demonstrated the best outcomes in certain SLE groups [118]. Lupuzor/P140 given at a dose of 200 µg thrice at 4-week intervals for 12 weeks was efficacious and generally well tolerated in SLE patients [119]. A peptidomimetic called FISLE-412 can neutralize anti-dsDNA autoantibodies linked to SLE. It has been demonstrated that FISLE-412 inhibits harmful interactions with tissue antigens and lupus autoantibody-mediated antigen recognition by serum from SLE patients [120].

A synthetic peptide called LJP-394 causes the immune system to become resistant to double-stranded DNA (dsDNA). It is a tetrameric oligonucleotide that has been shown to reduce lupus nephritic flare episodes. Thus, so far 14 clinical trials have examined LJP-394, and the results indicate that it is both safe and effective in lowering circulating anti-dsDNA antibodies and disease activity in individuals with active SLE [121]. Clinical studies on lupus have not shown much success with peptide-based treatments. In clinical studies, peptides have been demonstrated to be highly effective except for their safety and tolerability. Peptide-based therapies selectively target specific B-cell or T-cell populations. Despite this, peptide-based therapy has many challenges, starting from identifying the optimal peptide sequences along with their dosage [122]. Nanotechnology can aid in the proper delivery of peptides to a target, reducing toxicity and increasing efficacy [123]. The histone peptide shows remarkable tolerance for inhibiting immune cells and is nontoxic when specific nanoparticles are used. The potential risks associated with the peptide-based therapy include membrane imperiality and poor in vivo stability [124]. Zimmer et al. evaluated peptide Lupuzor which was well tolerated in SLE patients, the only minor adverse event was injection-site erythema, and in few patients pneumonia, soft-tissue infection, diverticulitis, and gastritis were observed [125]. Peptides are often rapidly degraded by enzymes in the body, limiting their bioavailability and efficacy. This necessitates frequent dosing, which can be inconvenient and costly for patients. Peptides may bind to unintended targets, leading to off-target effects and potential toxicity. Responses to peptide therapy can vary significantly among individuals, making it difficult to predict outcomes. Tailoring treatment to individual patients may be necessary to maximize efficacy. Peptides have shown safety and tolerability in clinical trials, but only a small number have shown effectiveness in reducing disease activity in SLE. This poses a challenge in developing appropriate biomarkers for monitoring response and exploring combination therapies involving peptides and other immunosuppressants for future clinical use [121]. In conclusion, despite the drawbacks of traditional immunosuppression, peptide-based treatments have emerged as novel immunomodulatory therapies for SLE (Tables 1 and 2).

**Table 1:** New Drugs in the Pipeline in the Field of SLE

Drug	Trial	Target	References
CNTY-101	Phase I approved by the FDA	CNTY-101 uses iPSC-derived natural killer cells with CD19-directed CAR, targeting CD8 + T cells, CD4 + T cells and NK cells	https://investors.centurytx.com/node/8076/pdf
Deucravacitinib	Phase II completed	Deucravacitinib inhibits tyrosine kinase 2 inhibitor binding to the pseudo kinase domain	Morand E, Pike M, Merrill JT, et al. Deucravacitinib, a Tyrosine Kinase 2 Inhibitor, in Systemic Lupus Erythematosus: A Phase II, Randomized, Double-Blind, Placebo-Controlled Trial. *Arthritis Rheumatol*. 2023;75(2):242–252. https://doi.org/10.1002/art.42391
Orelabrutinib	Phase Ib /IIa completed	Covalent inhibitor of Bruton’s tyrosine kinase	Mok CC. Targeted Small Molecules for Systemic Lupus Erythematosus: Drugs in the Pipeline. *Drugs*. 2023;83(6):479–496.https://doi.org/10.1007/s40265-023-01856-x
Saphnelo	Phase III completed	Type I receptor antagonists	Tanaka Y. Viewpoint on anifrolumab in patients with systemic lupus erythematosus and a high unmet need in clinical practice [published correction appears in RMD Open. 2024 Apr 4;10(2):]. *RMD Open*. 2023;9(3):e003270. https://doi.org/10.1136/rmdopen-2023-003270
JNJ-55920839	Phase I completed	Neutralizes IFNα subtypes and IFNω	Jordan J, Benson J, Chatham WW, et al. First-in-Human study of JNJ-55920839 in healthy volunteers and patients with systemic lupus erythematosus: a randomized placebo-controlled phase 1 trial. *Lancet Rheumatol*. 2020;2(10):e613-e622. https://doi.org/10.1016/S2665-9913(20)30223-X
Atacicept	Phase II completed	Binds to BAFF and APRIL	Wallace DJ, Isenberg DA, Morand EF, et al. Safety and clinical activity of atacicept in the long-term extension of the phase 2b ADDRESS II study in systemic lupus erythematosus. *Rheumatology (Oxford)*. 2021;60(11):5379–5389. https://doi.org/10.1093/rheumatology/keab115

**Table 2:** New therapeutic avenues for systemic lupus erythematosus

Therapy/Technique	Identification of the target/mechanism	Current status
CAR-T-cell therapy	In SLE CD -19 B are targeted through activated T cells, with the mechanism to directly kill targeted cells without the need for any accessory cell type	FDA-approved CABA- 201 for the treatment of SLE and Lupus Nephritis. It depletes CD19-positive B cells KYV-101 CAR-T cells with lymphodepletion conditioning in refractory lupus nephritis class IV with 12 patients are undergoing ongoing open-label phase trial 1
Single-cell transcriptomics	Identify cell type-specific molecular and genomic signatures in SLE Type I interferon pathway revealed from most of the transcriptomics study	Anifrolumab has shown promising results in active lupus nephritis. The drug is in phase 2 trial targeting specifically type I interferon receptor and blocking signaling pathway which is known to be involved in lupus nephritis
Transcriptomics	Characterize gene pathways and pathogenic drivers in lupus through an algorithm based on transcriptomic gene network JAK-STAT signaling in SLE is a key player	Baricitinib targeting JAK 1 and 2 is in phase 3 study showed successfully fulfilling primary endpoints but not secondary endpoints
Stem Cell	Mesenchymal stem cells act as both innate and adaptive immune pathways. They affect macrophages, monocytes, and dendritic cells (DCs) by suppressing the proliferation of CD4 and CD8 T lymphocytes and promoting the proliferation and differentiation of regulatory T cells (Treg)	Phase I trial of umbilical cord mesenchymal stromal cells in patients with treatment-refractory systemic lupus erythematosus showed promising and minimal adverse effects. The mechanistic study reveals. The B-cell changes and the GARP-TGFβ increase and correlate with SLEDAI scores with the safety of infusion in the patients
Therapeutic peptide	A synthetic-derived peptide which acts as an immunomodulator rather than an immunosuppressor. They target autophagy-mediated pathways and deplete hyperactive B-cell and T-cell pathways	Edratide in the phase II trial in patients with active SLE was found to be safe and well tolerable to the patients. However, some endpoints need additional evaluation to be included in the standard regimen Lupuzor/P140 is in phase IIb trial shown better efficacy and SLE responder index with only adverse events of erythema

### Small molecules as therapeutic targets

Understanding the pathogenesis of SLE is challenging; however, novel small molecules may reverse the pathophysiology of the disease by targeting intercellular signaling pathways. The targeted molecules are convenient both in terms of administration and affordability and lack immunogenicity [126]. Janus kinases, Bruton’s tyrosine kinase, immunoproteasomes, etc., have shown potential in phase II/III trials for SLE, indicating that small molecules hold promise for personalized therapy in the future (PMID: 36972009).

Small molecules target several pathways, such as Janus kinases, Bruton’s tyrosine kinases, and spleen tyrosine kinases, and activate downstream signals from immune cells that regulate various cytokines, chemokines, hormones, and B-cell receptors. With advancements in technology, these small molecules will be new drivers in the treatment of systemic lupus erythematosus and will contribute to the development of precision medicine [127, 128].

Toll-like receptors (TLRs) are important for understanding the host mechanism and are involved in the pathophysiology of lupus through their ability to recognize self-molecules. Numerous TLRs in humans and animals, such as TLR2/4, TLR5, TLR3, and TLR7/8/9, have been linked to the pathophysiology of SLE. MyD88, IRAKs, and IFN-α are examples of TLR signaling cascades that have been identified as possible therapeutic targets for the treatment of SLE [129]. Furthermore, the recently discovered novel modification of the TLR signaling system by long-coding RNA regulation and microRNA holds promise for SLE therapy in the future [130]. Six small molecules—acetohexamide, suloctidil, terfenadine, prochlorperazine, mefloquine, and triprolidine—have the potential to cure lupus nephritis, according to research performed by Qing et al. utilizing a bioinformatics approach [131]. Povetacicept (ALPN-303) is an Fc fusion protein that has demonstrated encouraging outcomes in preclinical models of SLE. It functions mainly by decreasing APRIL and BAFF [132]. A recent study published in Nature Chemical Biology showed that targeting the SLC15A4 protein is a potential drug avenue specific for endolysosomal TLR stimulation by the molecule AJ2-30 [133]. Lack of evidence on the difference in efficacy and safety among different targeted small-molecule drugs used in SLE. Meta-analysis conducted by Wang et al. showed that the adverse reactions of targeted small-molecule drug therapy are mild, and symptoms include nausea, vomiting, urinary tract infection, and upper respiratory tract infection [134]. Small molecules have shown promise in targeting specific pathways involved in SLE, offering potential therapeutic benefits. Over time, pathogenic cells can develop mutations that confer resistance to small molecules. Small molecules are more economically sustainable and accessible than biologics [135].

## Conclusions

The clinical and molecular heterogeneities of SLE are well accepted. The newer technologies developing in this field may help in the stratification of patients in various disease subtypes. Additionally, targeting certain pathways will also facilitate tailored therapy for treatment. Groundbreaking diagnostic developments in the field of molecular and genomic research are evolving to predict the lupus flare, creating a new unexplored path. Techniques like miRNA and single-cell transcriptomics will identify precise cell types to open new avenues in drug design.

Detection of risk loci may comprehend patients’ unique genetic makeup and understand the evolutionary mechanism of the disease. Newer technologies like pharmacogenomics will help identify the response of the drugs and their targeted organ. CAR-T-cell therapy already revolutionized this field, but its long-term effect and cost-effectiveness raise a question. The various therapeutic peptides are in queue, and different phases of clinical trials show interesting targets down the road. The use of hematopoietic stem cell therapy in daily practice remains challenging due to dysregulated immune systems and posttransplant complications. However, small molecules prove better in terms of cost-effectiveness and immunogenicity in comparison with traditional treatment regimens.

Combinatorial diagnostics and the evolution of many newer techniques are at an experimental stage. The various drugs are in the discovery phase, and some are already in the last stages of clinical trials. The future lies in the development of newer technologies working specifically, targeting, and understanding more meticulously the molecular mechanism of the disease. Given the complexity of this disease along with its multigenic risks, deeper investigations are imperative to come up with a remedy. As of now, the known knowns are the strong phenotype of this disease that rheumatologists can deal with. The known unknowns are the clinical trials and novel therapeutic discoveries and their outcome. The unknown are the drug specificities, collateral damages, and emergence of drug resistance. A multiomics-based systems biology approach seems to be the way to take it forward.

## Data Availability

No datasets were generated or analyzed during the current study.
